# Ataxia, intentional tremor and hypotonia syndrome caused by a novel *POU4F1* gene mutation: a case report

**DOI:** 10.3389/fgene.2025.1702803

**Published:** 2025-10-14

**Authors:** Qisheng Hu, Feng Zhu, Wanfen Wang, Yihang Xu, Haiyan Ren, Yanni Zheng, Yiqing Jiang, Shaofa Ke

**Affiliations:** ^1^ Department of Neurology, Taizhou Hospital of Zhejiang Province Affiliated to Wenzhou Medical University, Taizhou, China; ^2^ Department of Radiation Oncology, University of Miami Miller School of Medicine, Miami, FL, United States

**Keywords:** ataxia, hypotonia, Pou4f1, case report, hypokinesia

## Abstract

Childhood-onset ataxia, intention tremor and hypotonia syndrome (ATITHS) is a rare neurological disorder that encompasses features of hereditary ataxia, hypotonia. To date, only one report has associated the pathogenic variant in the POU4F1 gene with ATITHS. We report the case of a 28-year-old male who presented with lifelong gait instability and hypokinesia. The brain magnetic resonance imaging of this patient revealed significant cerebellar atrophy. Genetic analysis identified a novel heterozygous nonsense variant in Pou structural domain class 4 transcription factor 1 (*POU4F1*), which is predicted to result in loss of normal protein function. Segregation analysis within the family confirmed the presence of this variant in multiple symptomatic relatives. We confirmed diagnosis of ATITHS for this patient. This report provides additional evidence linking this mutation to specific neurologic disorders. We emphasize the importance of genetic testing to determine genetic etiology in patients presenting with ATITHS.

## Introduction

Childhood-onset ataxia, intention tremors and hypotonia syndrome (ATITHS) is a rare neurologic disorder characterized by features of hereditary ataxia and hypotonia. It is proposed that the heterozygous loss-of-function (LOF) variant in *POU4F1* is the causative agent of this novel ataxia syndrome (Webb et al., 2021).

*POU4F1*, also known as *BRN3A*, encodes a class IV POU domain–containing transcription factor (Herr et al., 1988). Its associated phenotype is primarily expressed in the nervous system and plays a key role in neuronal development. The gene produces two forms: a long form that contains an additional 84 amino acids at the N-terminus and a short form lacks this region. The C-terminal POU domain, common to both forms, activates a number of other neuron-expressed genes that stimulate neuronal growth (Latchman, 1998).

To our knowledge, only one report has associated the pathogenic variant in the *POU4F1* gene with ATITHS. It reported four independent patients with ataxia, intention tremor, and hypotonia, identified 4 *de novo*, heterozygous, LOF variants in *POU4F1*(Webb et al., 2021).

In this study, we applied the Whole Exome Sequencing (WES) and Validated Sanger Sequencing Analysis to identify pathogenic mutations in a Chinese patient with walking instability. We report a 28-year-old man with a history of lower limb weakness and gait instability since childhood. Genetic analysis revealed a novel heterozygous nonsense variant in the *POU4F1*. Analysis of the family segregation prompted its reclassification as pathogenic. This is the first reported case of a nonsense variant of *POU4F1* associated with this ataxia syndrome. It is also the first report of ATITHS involving a multigenerational pedigree.

## Case presentation

A 28-year-old Han Chinese male was admitted to Taizhou Hospital with a lifelong history of gait instability. Dizziness with intermittent nausea has been reported over the past year. The patient had been hypokinetic and developmentally delayed since childhood, with poor performance in physical education, He did not begin walking until the age of six and was unable to run long distances and jump high during childhood. The patient’s father informed us that the patient had a tremor as a child, which disappeared in adulthood. In adulthood, he continues to experience unsteady gait and difficulty with weight-bearing ambulation. On examination, he showed fair physical development, weight 65kg, height 163cm, along with concurrent cognitive impairment and poor learning ability.

**FIGURE 1:**
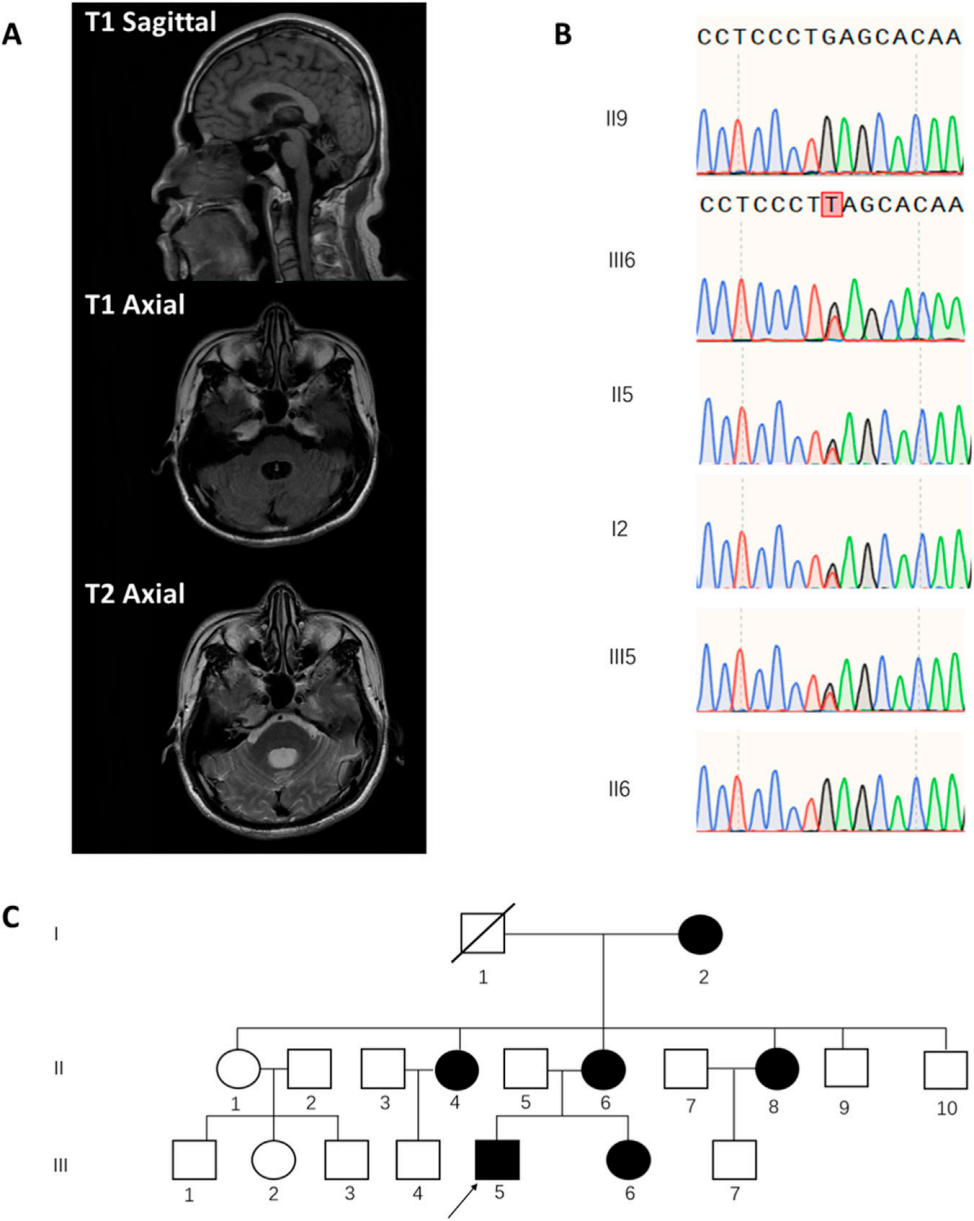
Clinical Imaging, Genetic Analysis, and Pedigree of a Patient with ATITHS Carrying a POU4F1 Nonsense Variant. **(A)** T1-and T2-weighted MRI showing dilatation of the fourth ventricle and cerebellar atrophy. **(B)** Sanger sequencing chromatograms showing the heterozygous nonsense variant, *POU4F1*. NM_006237.4:c.55G>T in the proband (III5), his sister (III6), mother (II6), and grandmother (I2), but absent in his healthy father (II5) and maternal uncle (II9). **(C)** Family pedigree chart showing segregation of the *POU4F1* variant. (arrow indicates the proband).

In the physical examination, the patient exhibited a wide-based gait and positive linear tandem walk test (Supplementary Video S1). Examination may reveal a positive heel-to-shin test, suggesting possible cerebellar ataxia. Tendon reflexes were normal, and Babinski sign was negative. Mild bilateral calf muscles atrophy was observed, with increased flaccidity during movement. We noticed hypotonia of the lower limbs. However, muscle strength was preserved in all four limbs. Notably, the patient demonstrated abnormal ocular motility, characterized by impaired up and down vision and abduction in both eyes (Supplementary Video S1). Intelligence testing revealed cognitive impairment, with an MMSE score of 17 and a MoCA score of 12. Further neuropsychological testing demonstrated deficits in processing speed, working memory, situational memory, visuospatial abilities, and verbal comprehension. Magnetic resonance imaging (MRI) showed dilatation of the fourth ventricle and cerebellar atrophy as shown in Figure 1A. The patient’s electroencephalogram (EEG) was within the normal range.

A family survey revealed that the patient’s mother, sister, and maternal grandmother exhibited similar symptoms, including unsteady gait, bilateral knee inversion, and difficulty in weight-bearing ambulation. His sister and mother presented with noticeable ophthalmologic abnormalities, while sister underwent esotropia surgery 2 years ago. Additionally, the patient’s two maternal aunts were reported to have similar symptoms (Figure 1C for the family pedigree chart). Medical examinations and genetic sequencing could not be performed on all relatives owing to difficulties in obtaining informed consent from some family members.

To determine the genetic basis of this patient’s condition, we performed whole exome sequencing (WES) on DNA extracted from peripheral blood. We identified a heterozygous nonsense mutation, the *POU4F1,* NM_006237.4:c.55G>T(p.Glu19*) variant, The WES results were then validated by direct Sanger sequencing analysis. This variant leads to loss of normal protein function through nonsense-mediated mRNA decay or premature termination of amino acid translation. The pathogenicity of other LOF variants in *POU4F1* has been previously reported and is supported by databases such as ClinVar and HGMD, fulfilling the ACMG PVS1 criterion. Additionally, this variant has not been previously reported in the literature or listed in the large population frequency database such as gnomAD, meeting the ACMG PM2 criterion. The absence of this variant in the peripheral blood DNA of the proband’s asymptomatic father supports its maternal inheritance and strengthens the evidence for segregation with disease within the maternal lineage.

We then performed Sanger sequencing analysis on peripheral blood DNA from the proband’s mother and asymptomatic maternal uncle, as well as hair follicle DNA from the sister and grandmother. The same variant was detected in the symptomatic mother, sister, and grandmother, but was absent in the healthy uncle in the same family (Figure 1B) which is consistent with ACMG evidence PP1. Considering the above results and in accordance with the ACMG guidelines, we classify the *POU4F1,* NM_006237.4:c.55G>T (p.Glu19*) variant classified as pathogenic, supported by evidence PVS1, PM2 and PP1.

Thus, the combination of clinical presentation and genetic analysis supports the diagnosis of ATITHS in this patient, associated with the *POU4F1* c.55G>T (p.Glu19*) mutation. Although he is currently able to complete basic daily activities with intermittent muscle strength and balance rehabilitation, his weight-bearing remains reduced compared to healthy individuals. Given the likely pathogenic nature of this variant and its familial segregation, we recommend genetic counseling and prenatal diagnosis for future pregnancies in this family to prevent potential transmission of the mutant allele.

## Discussion

ATITHS is a rare neurodevelopmental disorder inherited in an autosomal dominant manner, characterized by childhood-onset ataxia, intention tremor, and hypotonia, often resulting in delayed ambulation. Additional clinical features may include global developmental delay, mildly impaired intellectual development, speech delays or learning disabilities, and abnormal eye movements. The disease is rarely reported, and its full phenotypic spectrum is still being defined. Pathogenicity of the *POU4F1* gene is believed to underlie the condition, consistent with its critical role in neuronal development.

Webb et al. first reported a pathogenic variant in *POU4F1* associated with ATITHS in four unrelated patients (Webb et al., 2021). All four patients in their report presented with motor symptoms like ataxia. In the oldest patient, obligatory paroxysmal head lifting diminished over time. A similar pattern was observed in our study: the patient had increased motor abilities in adulthood compared to early childhood. Interestingly, the patient in our study had persistent ocular motility symptoms into adulthood. Furthermore, similar eye movement symptoms were reported in his mother and sister. Although oculomotor palsy was not explicitly described in the earlier report, three of the patients had strabismus and underwent esotropia surgery, suggesting that partial oculomotor palsy may have been present but underrecognized. These findings indicate that ophthalmologic symptoms represent a key clinical feature in patients with ATITHS, especially in older patients and may serve as an important diagnostic clue of the disease. A comparison between the present case and previously reported cases is summarized in Table 1.

**TABLE 1:** Comparison of Clinical Characteristics Between the Present Case and Previously Reported ATITHS Patients with *POU4F1* Variants by Webb et al.

Clinical feature	Patient from this study	Patients from Webb et al.^1^			
		Case 1	Case 2	Case 3	Case 4
Variant	heterozygous	heterozygous	heterozygous	heterozygous	heterozygous
	NM_006237.4:c.55G>T(p.Glu19^a^)	c.917A>G; p.Gln306Arg	c.158_161dup; p.Leu55AlafsTer295	c.283_290del; p.Thr95SerfsTer251	c.271_281del; p.Thr91HisfsTer254
Gender	Male	Female	Male	Male	Male
Age at exam	28 years	3 years 5 months	3 years, 10 months	4 years, 2 months	22 years
Birth history	via vaginal delivery	via C-section	via vaginal delivery	via vaginal delivery	via vaginal delivery
Ataxia Symptoms	Present	Present	Present	Present	Present
Hypotonic	Present	Present	Present	Present	Present
Cognitive function	Mild cognitive delays	Global developmental delay	Global developmental delay	Global developmental delay	Isolated motor delays from infancy; learning disability/mild cognitive delays noted at older age
Ocular motility abnormalities	Impaired up and down vision and abduction	None appreciated (Left eye esotropia s/p^a^ strabismus surgery)	None appreciated	None appreciated (Left eye esotropia s/p surgery at 14 months and 39 months)	Spontaneous drifts of the eyes upwards superimposed on tonically held upgaze(Bilateral esotropia s/p surgery)
Developmental Milestones	Walked at 6 years	Rolled over at 8 months in both directions; sat at 10 months; crawled at 14 months walked at age 2 years	Walked at 3 years 1 month	Rolled over at 9 months in both directions; sat at 9 months; crawled at 2.5 years; at 4 years is able to walk with a gait trainer	Rolled over in both directions at 18 months; sat at 3 years; walked independently at 8–10 years
MRI	dilatation of the fourth ventricle and cerebellar atrophy	None appreciated	None appreciated	None appreciated	The inferior olivary nuclei show prominent symmetrical signal increase at age 11.2 months and age 11.0 years that fades by age 16.4 years; progressive vermian atrophy
Family history	Mother/grandmother/sister	-	-	-	-

^a^ s/p: status post.

Eye movement disorders are commonly attributed to peripheral neuropathy, while certain intracranial lesions can also cause impaired ocular motility (Musazadeh et al., 2004; Williams and Hoyt, 1989; Schreuders et al., 2012). Several brain regions are involved in the control of normal eye movements. The neural pathways responsible for smooth pursuit eye movements are believed to project to the cerebellum and then to the premotor regions of the brainstem after integration through the pontine nuclei. Both the cerebellar pons and the parvocellular pons receive inputs from the pontine nuclei, with the ventral parvocellular pons playing a particularly important role in smooth pursuit eye movements (Nagao et al., 1997; Rambold et al., 2002). In addition, other cerebellar structures, such as the oculomotor vermis (OMV) and the fastigial nucleus oculomotor (FOR), are also involved in maintaining smooth ocular tracking (Heinen and Keller, 1996). A range of eye movement symptoms observed in patients with ATITHS may be related to cerebellar atrophy involving these regions.

In this case, the patient did not exhibit significant palatal tremor at the time of examination at age 28. Neuropathologically, in most cases of palatal tremor, the lesion originates in the central pericentral fasciculus, which connects the red nucleus and the deep cerebellar nucleus to the inferior olive (Hainline et al., 2017). As reported in a previous study, serial MRI scans highlighted the disease progression, showing marked symmetric T2 hyperintensity in the inferior olive nucleus at younger ages, which tends to fade over time, while cerebellar atrophy progressively became more pronounced (Webb et al., 2021). Since our patient was first examined at age 28, the dominant MRI findings were cerebellar atrophy. Due to the lack of earlier imaging data, the longitudinal relationship between MRI changes and clinical features remains uncertain.

*POU4F1* plays a critical role in the development of the sensory peripheral nervous system. In animal model, affected *Pou4f1* ^*−/−*^ neurons undergo apoptosis and are unable to properly innervate their peripheral target organs. This leads to loss of other functions in sensory axon growth (Eng et al., 2001; Xiang et al., 1997), which may further result in uncoordinated limb movement and impaired sucking in mice after birth (Xiang et al., 1996; Agarwala and Ragsdale, 2002). Its role in the central nervous system, particularly in regions such as the parietal nuclei, reinnervating nuclei, and the retina, has also been extensively investigated (Fedtsova et al., 2008; Badea et al., 2012; Quina et al., 2009).

*Pou4f1* is expressed in the red nucleus (RN) during both embryonic development and adulthood, and it defines the RN signature. Deletion of *Pou4f1* in mice leads to disorganization of the rubrospinal tracts (Martinez-Lopez et al., 2015), a system that plays an important role in the development of basic motor functions.

In conclusion, our study describes a male patient with adolescent peripheral neuropathy carrying the heterozygous nonsense mutation *POU4F1* NM_006237.4:c.55G>T(p.Glu19*) chr13:79177407. As the first reported case of ATITHS with a complete family lineage, our study has described more neurological features of this particular neurological syndrome. Our findings further support a causal relationship between this novel mutation and ATITHS, contributing to its classification to pathogenic. We emphasize the importance of genetic testing to determine genetic etiology in patients presenting with ATITHS, which can significantly improve diagnostic accuracy, guide clinical management and inform genetic counseling for affected families.

## Data Availability

The datasets presented in this article are not readily available because of ethical and privacy restrictions. Requests to access the datasets should be directed to the corresponding authors.
